# Core body temperatures during final stages of life—an evaluation of data from in-hospital decedents

**DOI:** 10.1007/s00414-022-02837-7

**Published:** 2022-06-11

**Authors:** Patrick Scheidemann, Holger Schwender, Stefanie Ritz-Timme, Detlef Kindgen-Milles, Benno Hartung

**Affiliations:** 1 Institute of Legal Medicine, University Hospital Düsseldorf, Düsseldorf, Germany; 2 Mathematical Institute, Heinrich Heine University, Düsseldorf, Germany; 3 Department of Anesthesiology, University Hospital Düsseldorf, Düsseldorf, Germany; 4 Institute of Legal Medicine, Ludwig Maximilians University, Munich, Germany

**Keywords:** Death time, Estimation, Temperature, Agony, Influence factors, Nomogram

## Abstract

Temperature-based methods are widely accepted as the gold standard for death time estimation. In the absence of any other information, the nomogram method generally assumes that a person died with a core body temperature of approximately 37.2 °C. Nevertheless, several external and internal factors may alter the body temperature during agony. A retrospective medical record analysis was carried out on in-hospital death cases from two consecutive years of surgical intensive care units to determine the effects of factors influencing the core body temperature at the point of death. Data from 103 case files were included in the statistical data evaluation. The body temperature fluctuated between and within individuals over time. No clear correlation to certain death groups was observed. Even primary cardiac deaths showed broad intervals of temperatures at the point of death. Men seem to die with higher body temperatures than women. The presented data highlight potential biases for death time estimations when generally assuming a core body temperature of 37.2 °C. In conclusion, the estimation of the time of death should include various methods, including a non-temperature-dependent method. Any uncertainties regarding the body temperature at point of death need to be resolved (e.g. by identifying fever constellations) and elucidated if elimination is not possible.

## Introduction

The time since death estimation can be of elementary importance for the investigation procedure. The gold standard approach relies on observations of body cooling behaviour, which follows scientific principles.

As early as 1829, Davy described his observations on the temperature of the human body after death. He measured temperatures from 82 to 113 °F (about 27.8 to 45 °C) between 3 and 29 h post mortem [1]. Individuals exhibited pre-death fevers, so it was assumed that the cause of death affected the temperature at the time of death, which lasted into the post mortem period. Davy developed the first ideas on the use of post mortem body temperature: “It may often be a question, how long a body has been dead. By attention to its temperature, particularly of the deep-seated parts, taking into consideration the circumstances affecting temperature, probably most instances an answer may be given approximating to the truth and which may be of considerable use in evidence”.

Two main temperature-dependent methods are available for forensic purposes. The nomogram method is most widely used as it can be applied fast and easily [2–5]. Additionally, the finite element method [6, 7] was introduced which is far more complex to apply. A recently described thermodynamic finite difference model based on skin thermometry has yet to prove itself in daily practice [8].

Unless explicitly stated otherwise, the nomogram method always assumes a body temperature of 37.2 °C at the time of death [2, 3, 5, 9–11].

However, the general assumption of a body temperature of 37.2 °C at the time of death is sometimes daring. The fact that core body temperatures may deviate from assumpted 37.2 °C is long known [1], and also a recent examination of 25 bodies with leading causes of death in a neurosurgical intensive care unit revealed core body temperatures at the time of death of between 33.8 and 40.3 °C [12].

In this respect, the question arises whether the baseline body temperature of a deceased person may have been subject to fluctuations during agony, and the diseases or external influences that may determine the body temperature at the time of death.

Since the temperature at the point of death has a crucial influence on the time since death estimation, especially during the first few hours after death [13], the following questions were addressed using a controlled clinical setting:

- At what core body temperatures do patients die?
- Is a core body temperature interval definable for specific groups of causes of death?
- Which medical procedures have which influence on core body temperature?

The study was approved by the ethics committee of the University of Düsseldorf (study number: 2019–480).

## Material and methods

### Medical data

In an effort to answer the questions above, a retrospective medical record analysis of patients who died in the surgical intensive care units of the University Hospital Düsseldorf in 2017 and 2018 was performed.

The following parameters from the last 24 h before death were recorded:

- core body temperature (recorded maximum hourly),
- age and sex,
- height and weight,
- cause of death and comorbidities,
- blood pressure and pulse (maximum hourly),
- haemoglobin concentration (maximum hourly),
- infusions, transfusions, administered medication, and
- medical measures (extracorporeal life support resp. ECLS, dialysis, surgery, urinary bladder rinsing).

The data had been recorded on curve sheets in handwritten form during treatment and were not accessible to machine evaluation. Vital signs including temperature were documented hourly in most cases. With a length of stay of more than 1 day, an average of 24 documented values was available.

The temperature of the patients was derived using a temperature probe via a bladder catheter of the type “Teleflex Rüsch Sensor”. According to the manufacturer, these sensors have an accuracy of + 0.1 °C and − 0.2 °C [14].

The cause of death (as documented in the death certificate) was assigned to different categories analogous to Preuß-Wössner et al. [15] (Table 1).

**Table 1:** Allocated death categories (as documented in the death certificate) (derived from [15])

Bleeding or bleeding complication
Septic multi-organ failure (e.g. pneumonia, peritonitis)
Cardiac genesis (e.g. myocardial infarction, cardiac insufficiency)
Circulatory failure (e.g. pulmonary embolism, peripheral arterial occlusion)
Liver failure (e.g. liver metastases, hepatic vascular disturbance)
Kidney failure (e.g. renal insufficiency)
Respiratory insufficiency (e.g. pulmonary metastases, pleura empyema)
Cerebral hypoxia (e.g. after near-drowning, after myocardial infarction)

### Statistical analysis

#### Differences between death categories

Depending on whether the normality and/or the homogeneous variance assumption of the one-way ANOVA (analysis of variance) were violated or not, a one-way ANOVA, a Welch ANOVA not assuming equal variances in the different groups, or a Kruskal–Wallis test was used to test whether the values of the considered factor differs between the different death categories.

Post hoc tests (*t*-test, Mann–Whitney test) were applied to analyse differences between the death categories.

#### Differences between age groups

Without considering the category of death, it was tested whether there was a difference in temperature at the time of death between the age groups 4–20, 21–40, 41–60, 61–80, and 81–98 years. For this purpose, the above-mentioned multi-group tests were used. Since there were only a few persons under the age of 40, which negatively affected the results of the tests, we performed two analyses: (1) all five above-mentioned groups were analysed and (2) only the age groups of 41–60, 61–80, and 81–98.

Since age and temperature were not normally distributed, Kendall’s tau was applied to analyse the correlation between age and temperature.

#### Differences between sexes

Excluding the category of death, it was tested whether there was a temperature difference at the time of death between the sexes. Since two groups were compared here, a *t*-test was performed assuming that the variances in both groups were identical.

## Results

In 2017 and 2018, 436 patients died in both intensive care units. Temperature measurements within the last 24 h post mortem of deceased persons, who were not externally warmed or cooled, were available in 149 cases.

A total of 284 deceased patients presented incomplete data that could be used to correlate body temperature with vital signs, intravenous administration of fluids, and haemoglobin.

Temperature measurements of non-cooled and non-warmed deceased persons within the last 24 h ante mortem were available in 149 cases.

One hundred five cases had temperatures records within the last 2 h before death. Since circulatory failure and kidney failure contained only one person each, these categories were not included in the analyses. If not stated otherwise, the remaining 103 cases were included in the final statistical evaluation.

**Fig. 1:**
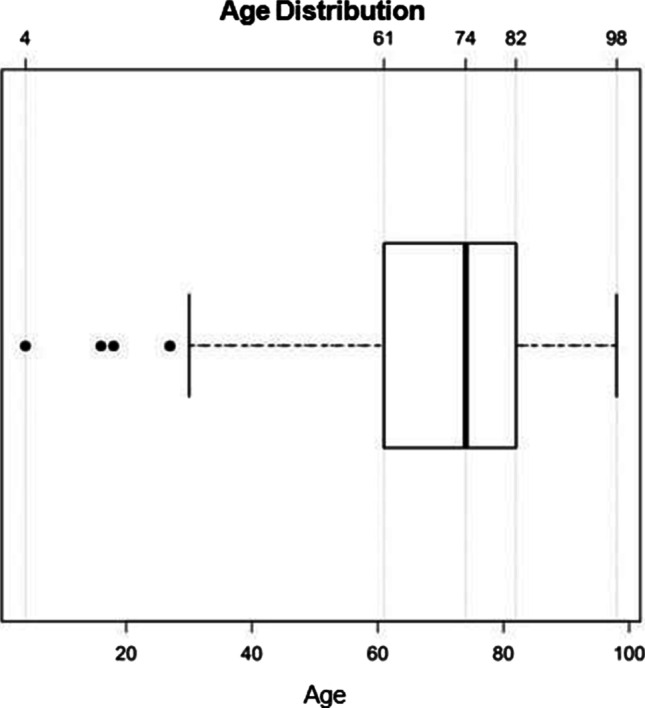
Age distribution (years) of the included deceased persons. Box contains 50% of the tested persons, the line inside the box indicates the respective median, and the satellites and individual outliers indicate 25% of the tested persons. Outliers are presented as dots. Total *n* = 103

Fifty percent of the deceased persons were 61 to 82 years old (median 74 years; range 4 to 98 years) (Fig. 1).

Among those 103 cases evaluated here, body weight was recorded in 97 cases (61 males, 36 females). Body height was recorded in 95 cases (59 males, 36 females). Median BMI was 26 for men (range: 13–45) and 24 for women (range 12–39).

No statistical significance was found between the body temperatures measured within a maximum of 2 h ante mortem in the above-mentioned five resp. three age groups (analysis of variance without assumption of equal variances: 0.27 resp. 0.26).

**Fig. 2:**
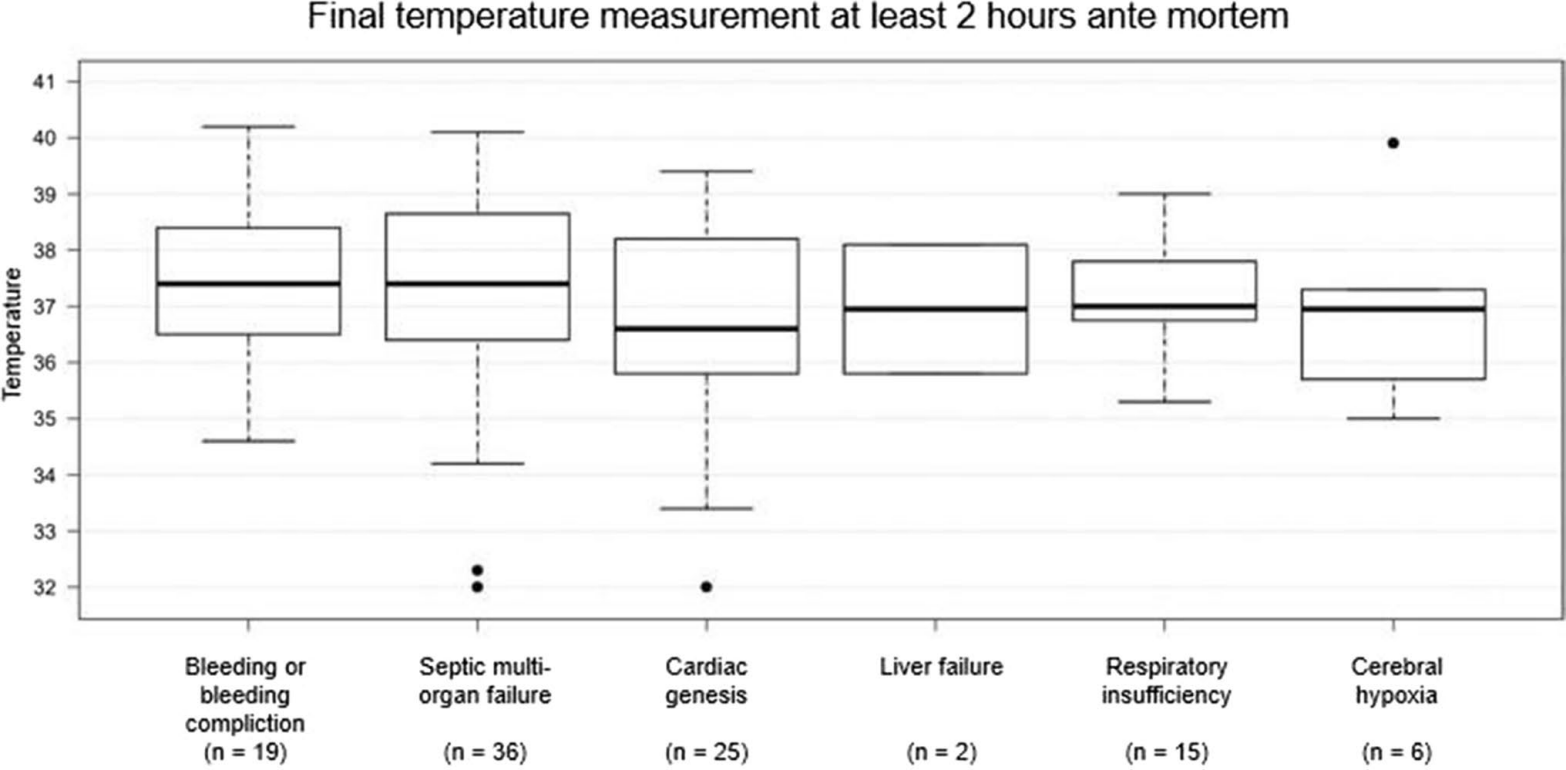
Death groups (x-axis), numbers of assigned deceased individuals (without external heating or cooling), and the final measured body temperature within 2 h ante mortem (°C, y-axis). Numbers are presented below each column. Total *n* = 103

Most causes of death were allocated to the group “septic multi-organ failure” (*n* = 36). Primary cardiac causes of death (“cardiac genesis”) made up the second largest group (*n* = 25). Bleeding resp. bleeding complications and pulmonary causes of death (“respiratory insufficiency”) represented other larger groups (*n* = 19 resp. 15) (Fig. 2).

The range of the last temperature measurements’ results within the last 2 h before death is presented in Fig. 2, too. The cardiac causes of death show a particularly wide range, especially towards subnormal levels. Bleeding or bleeding complications present very inhomogeneous temperatures at death, too.

**Fig. 3:**
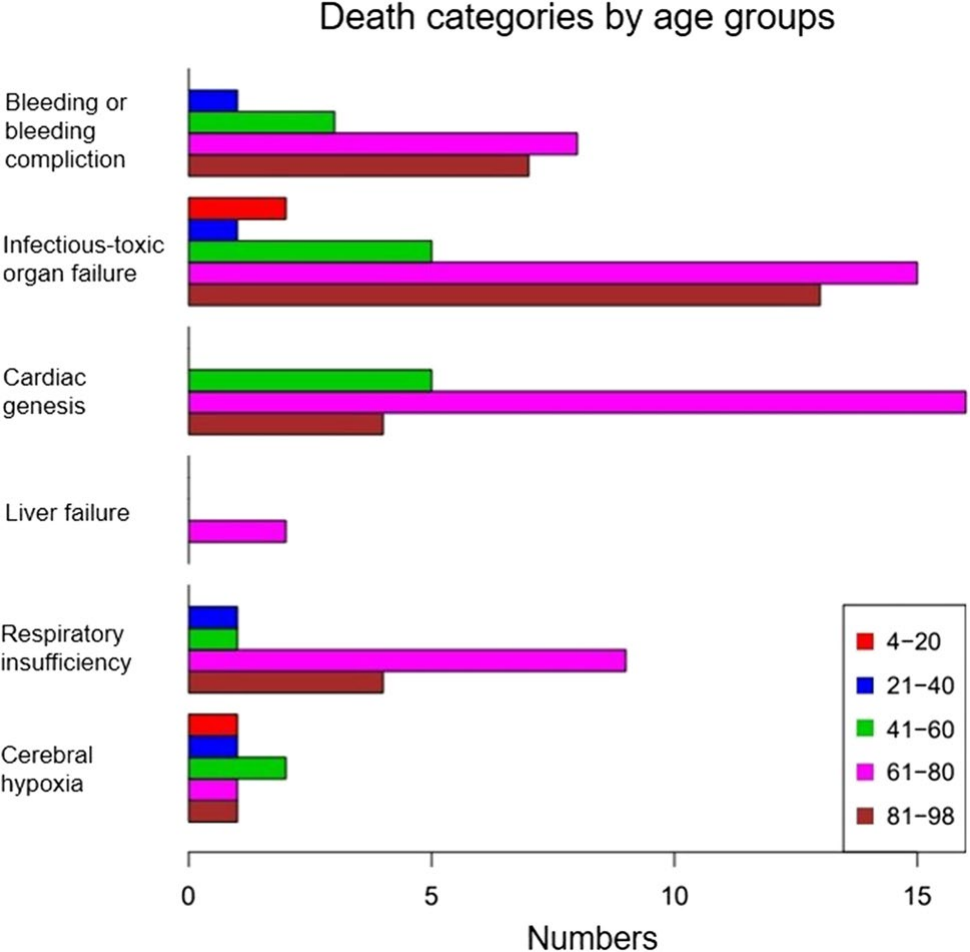
Death categories by age groups (number; x-axis). Red: 4–20 years, blue: 21–40 years, green: 41–60 years, pink: 61–80 years, and brown: 81–98 years

Very old persons (> 80 years) were overrepresented in the death group “septic multi-organ failure”, while seniors between 61 and 80 years were overrepresented in the “cardiac genesis” death group (Fig. 3).

There was no statistical significance between the different death categories and the body temperatures measured within a maximum of 2 h ante mortem (ANOVA: 0.61).

**Fig. 4:**
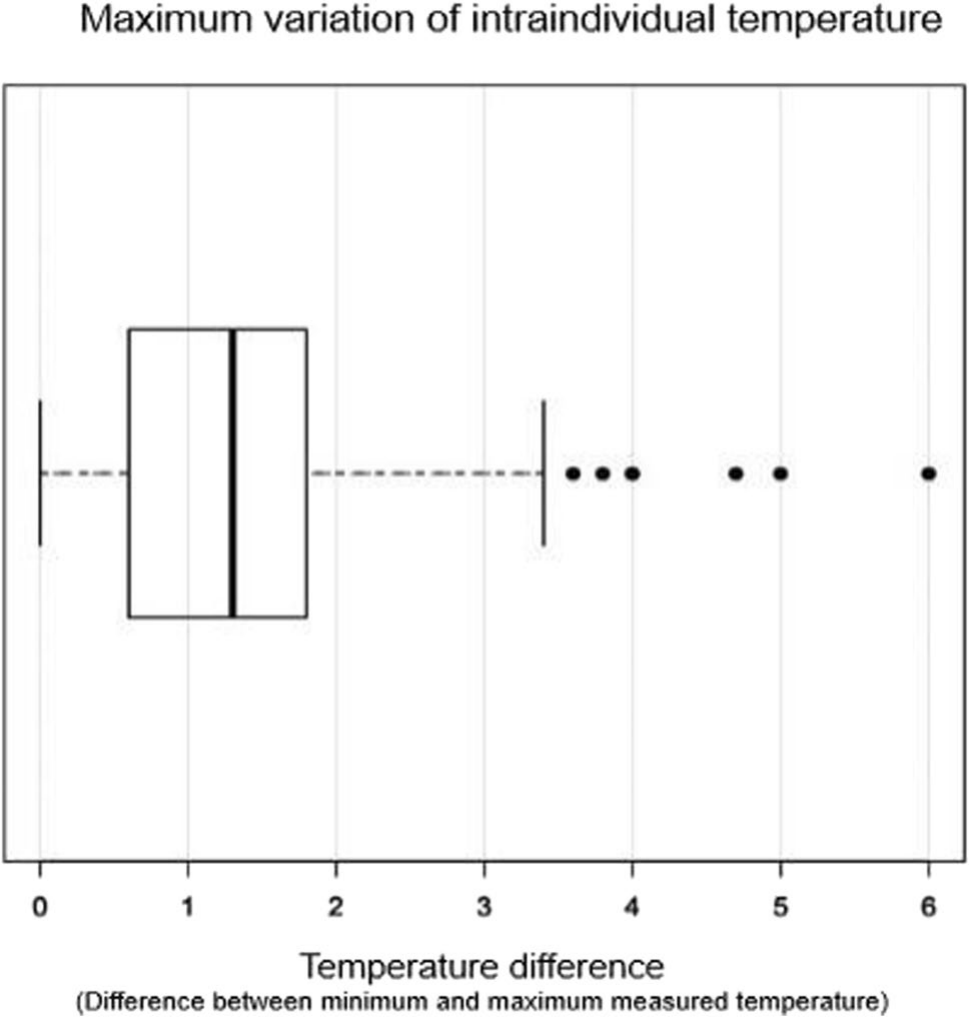
Intraindividual fluctuation of the body temperature (°C) during the last 24 h ante mortem. Total *n* = 149

Intraindividual fluctuations in body temperature regularly occurred. The median fluctuation during the last 24 h ante mortem was more than 1 °C (*n* = 149). Singular deviations of several degrees (up to 6 °C) occurred (Fig. 4).

**Fig. 5:**
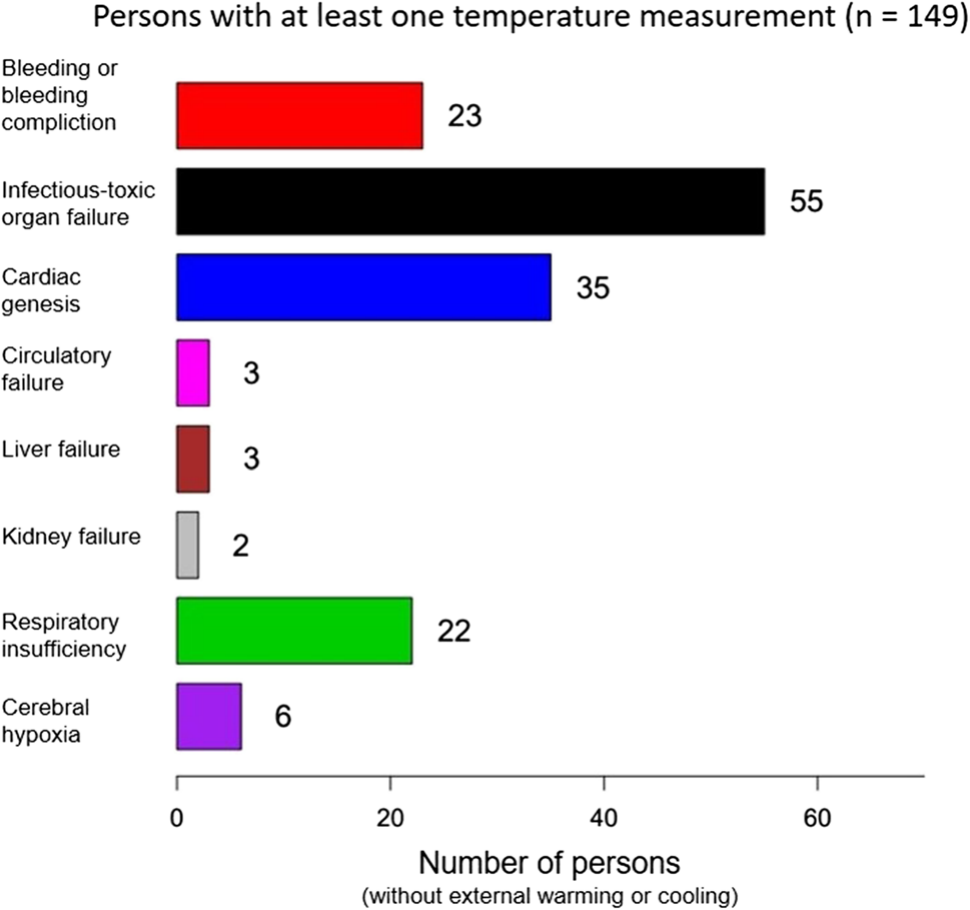
Number of persons in distinctive death groups with fluctuation of the body temperature of more than 1.5 °C in the last 24 h ante mortem

It is noteworthy that variations in body temperature of more than 1.5 °C (*n* = 52) were found in all groups, with the group of septic causes of death being obviously overrepresented (Fig. 5).

**Fig. 6:**
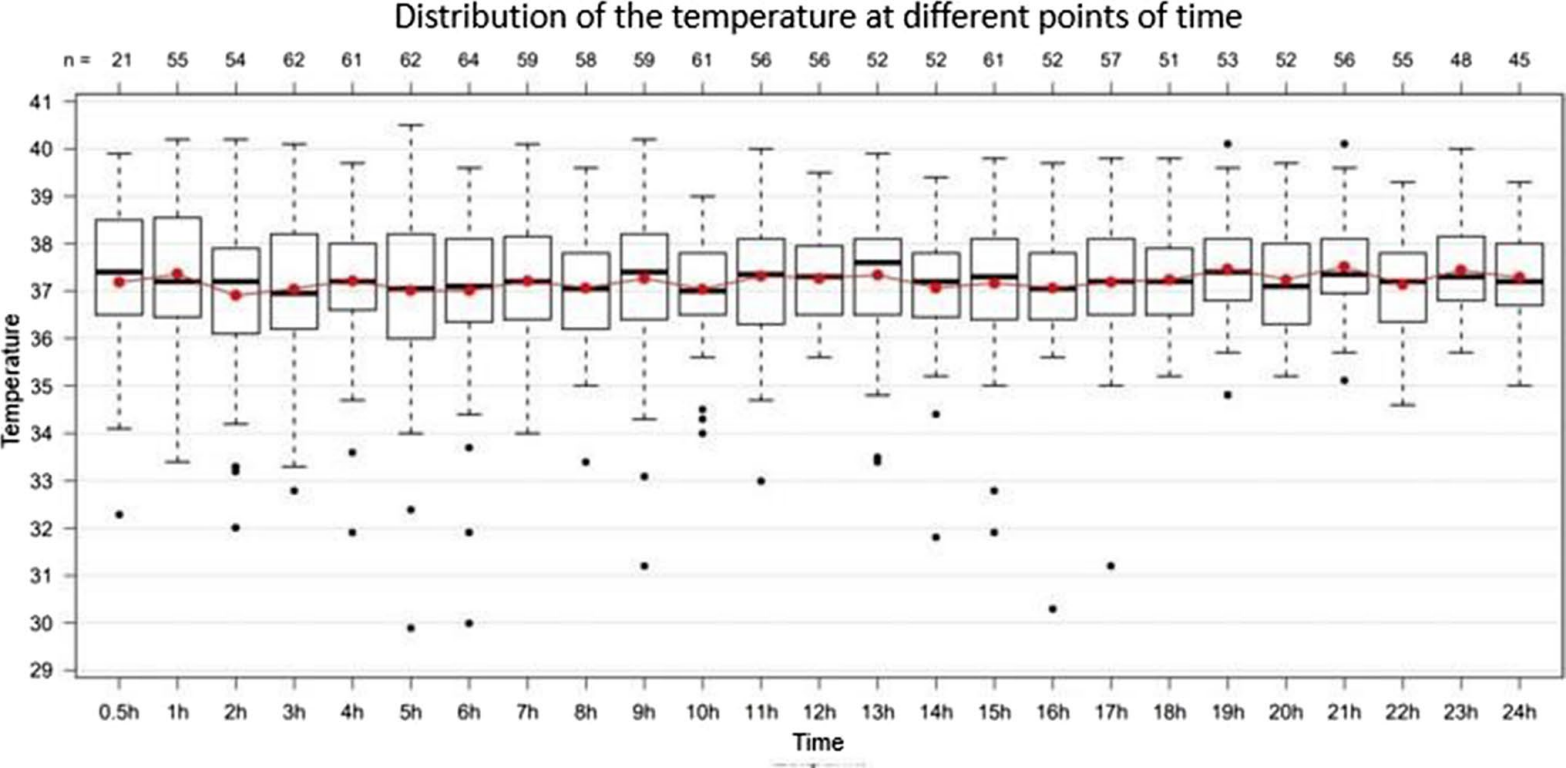
Fluctuation of the body temperature (°C; y-axis) during the last 24 h of life (hours ante mortem; x-axis). The red dots indicate average values. Total *n* = 103

The average core body temperature was mainly around 37.2 °C. The partially extreme interindividual fluctuations (spikes) must explicitly be pointed out here (Fig. 6).

**Fig. 7:**
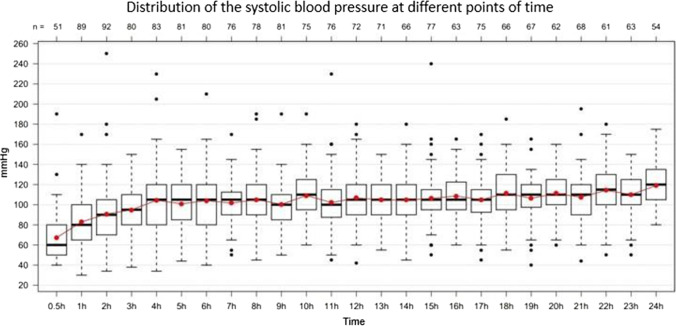
Fluctuation of the systolic blood pressure (mmHg; y-axis) during the last 24 h of life (hours ante mortem; x-axis). Total *n* = 103

**Fig. 8:**
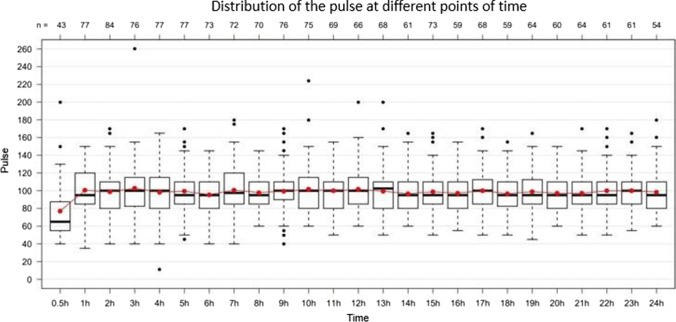
Fluctuation of the pulse (bpm; y-axis) during the last 24 h of life (hours ante mortem; x-axis). Total *n* = 103

Both systolic (Fig. 7) and diastolic blood pressure, and heart rate dropped significantly in the last hours before death. Regarding blood pressure, this effect could be observed within the last 3 h ante mortem, however, only within the last 1/2 h with regard to the heart rate (Fig. 8).

**Fig. 9:**
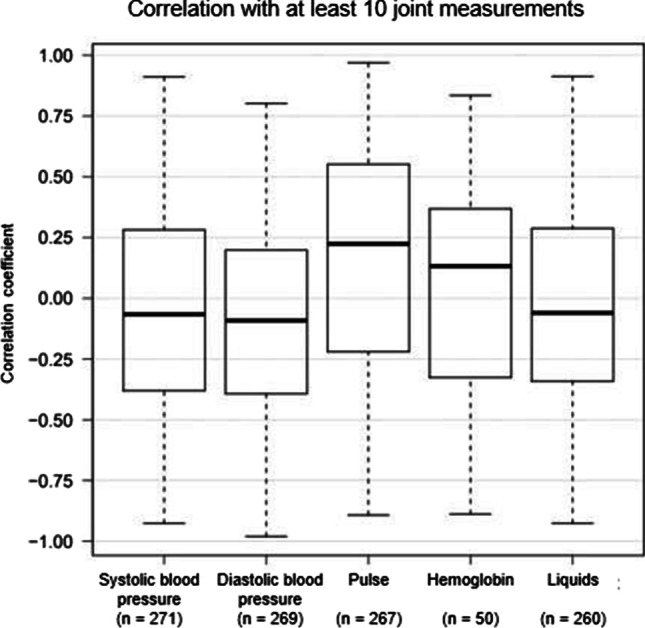
Correlation of vital signs, hemoglobin, and intravenously administered fluids (x-axis) with temperature in subjects who had at least 10 contemporaneous responses of temperature and the corresponding variable (*n* = 284). Data according to Pearson’s correlation coefficient (y-axis). The numbers of observations are presented below each column

When looking at the correlations of systolic and diastolic blood pressure, pulse, haemoglobin, and intravenously administered liquids with temperature using Pearson correlation coefficient, low positive and negative median correlations can be seen. For example, the pulse has a median correlation coefficient of about 0.25 with temperature (Fig. 9).

**Fig. 10:**
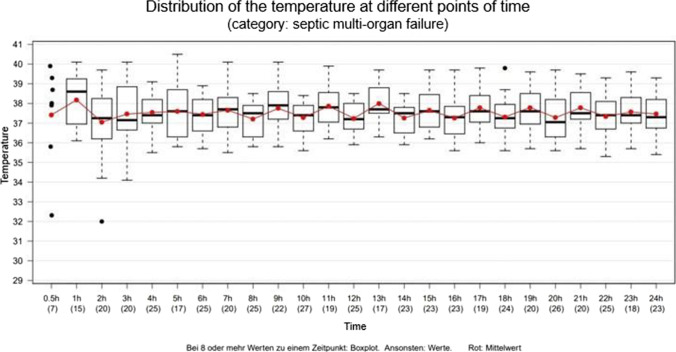
Fluctuation of the body temperature (°C; y-axis) during the last 24 h of life (hours ante mortem; x-axis). Total *n* = 36

When considering the death group “septic multi-organ failure” only, a broad range of body core temperatures can be seen, especially in the last few hours before death despite a controlled intensive care clinical setting with antipyretic and antibiotic medication (Fig. 10).

**Fig. 11:**
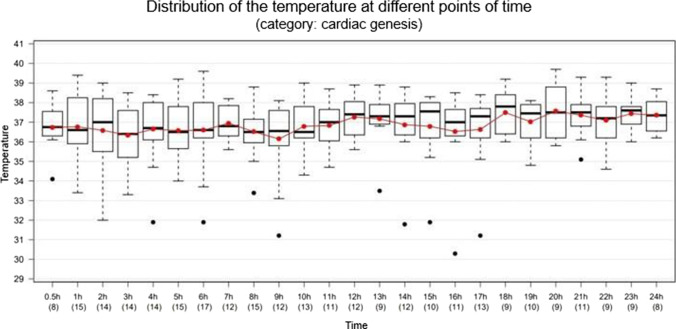
Fluctuation of the body temperature (°C; y-axis) during the last 24 h of life (hours ante mortem; x-axis). Total *n* = 25

The death group “cardiac genesis” shows a slight median decrease of body core temperature towards death (Fig. 11). Even though no fever constellations are included here, broad ranges are evident here, too.

Sex specific evaluations showed higher body temperatures in men than in women at the time of death (*t*-test for all temperatures obtained in the last 2 h ante mortem: 0.02; *n*_*male*_ = 170; *n*_*female*_ = 107).

The influence of therapy by ECLS or bladder irrigation on body temperature could not be addressed, because these therapies included external heating or cooling in most cases.

## Discussion

Core body temperatures may deviate from the assumed 37.2 °C prior to death [1, 12], and its effect on death time estimation has already been examined thermodynamically. It could be demonstrated that temperature biases affect death time estimation most intensely in the first few hours post mortem [13]. Muggenthaler et al. showed that at a body temperature of 41 °C at the point of death, a temperature of 37.2 °C (at an ambient temperature of 18 °C) is reached after about 5 h post mortem [13].

As it could be shown here, the core body temperature can rise as well as fall shortly before death. Considering all deceased persons, the average body temperature was around 37.2 °C which seems justified for in-hospital decedents, if externally warmed or cooled persons are excluded. However, the extreme interindividual fluctuations (spikes) must explicitly be emphasised here (Fig. 6). Of note, temperature measurements presented here were performed mainly in the urinary bladder. This measurement method is considered a standardised method for permanent temperature measurement in critically ill patients [16]. Lefrant et al. showed that rectal and bladder temperatures had a deviation of − 0.07 ± 0.40 °C and − 0.21 ± 0.20 °C, respectively, from pulmonary artery temperature [17]. Mravinac et al. also compared rectal and bladder temperature with pulmonary artery temperature. They found a higher correlation between pulmonary artery and bladder temperature than between pulmonary artery and rectal temperature [18]. Thus, there are generally minor differences between the two measurement methods. Nevertheless, bladder temperatures may be altered in patients with kidney failure and can lag behind body core temperatures during fast body core temperature alterations [19].

For the estimation of time since death estimation, it is paramount to identify factors that could affect core body temperature. Some authors estimated that approximately 10% of violent deaths are associated with hyperthermia [20]. Acute psychosocial stress (standardised laboratory stress task; Trier social stress test) is said to have a decreasing (sic!) effect on intestinal temperature, at least on upper gastrointestinal areas [21]. Acute psychosocial stress should be present in even more than 10% of all violent and thus forensically relevant deaths.

Obermeyer et al. [22] performed a meta-analysis of basal body temperatures that considered data from 35,488 living individuals who were neither suffering from infection nor had they been under antibiotic administration. The body temperatures were measured orally in 88.2%. The average temperature was 36.6 °C with a 95% confidence interval of 35.7–37.3 °C (99% range: 35.3–37.7 °C). The authors demonstrated that body temperature correlates with a range of demographic factors, accompanying comorbidities, and physiological conditions. Even the basal body temperature was correlated with mortality. Interestingly, body temperature was generally lower in summer than in winter months under comparable surrounding conditions (e.g. 0.08 °C difference between July and February). In line with these data from living individuals, it could be demonstrated that both blood pressure and pulse are slightly positively or negatively correlated with body temperature.

To avoid errors in Henssge’s temperature measurement method, Potente et al. suggest a procedure in which brute force calculations can be used to generate the parameters relevant to the calculations. This method may be helpful both scientifically and in practice in the future [23].

The influence of individual medications on body temperature could not be evaluated due to the many different medications and drug combinations administered on the intensive care unit.

The results presented here do not seem too promising to ease temperature-based death time estimation. Intra- and interindividual variations are immense, and they do not seem easily predictable, at least not for the presented death groups of surgical intensive care units.

Of course, one can raise the question to what extent the collective studied here reflects forensic reality. Thereby, it has to be kept in mind that at least two of the larger death groups (“cardiac genesis” and “bleeding/bleeding complications”) are common in the daily routine when death time estimation might be necessary. However, the finding is in line with Muggenthaler et al. who conducted a cooling study with 84 decedents. The spectrum of causes of death was said to be a typical subset of sudden death cases of forensic practice. The initial, partially delayed measurements of body temperatures varied between 31.1 and 38.7 °C. The authors highlighted that hypo- and hyperthermia occur quite regular in forensic death time estimation [24].

The results also indicate that men die with higher body temperatures than women do. As this finding is based on small observation groups, it should be re-evaluated in a more extensive data set. Gender-specific differences might at least be partially explainable by the different body weight resp. BMI. In our evaluation, median BMI was 26 for men (range: 13–45) and 24 for women (range 12–39).

## Conclusion

Limiting the possible interval of the deceased person’s body temperature at the time of death may be troublesome, but it is advisable in any case. Therefore, a first guess at the crime scene should be uttered with utmost restraint.

Medical history and results of histologic and toxicological examinations should be known for providing a second, more reliable guess. Histologic examinations might reveal fever constellations, and both medical history and toxicologic examinations might indicate temperature-affecting medication or diseases.

Generally, estimation of time since death should not be based on a single but different methods.

## Limitations

The work is limited by three factors in particular.

First, the division into groups had to be solved pragmatically with regard to the nature of death. Some cases could with some justification also be classified in other groups (e.g. cerebral hypoxia after myocardial injury could also have been attributed to “cardiac genesis”).

Second, we studied hospital descedants only. Therefore, it is questionable whether and to what extent our conclusions are valid for typical forensic cases outside the hospital.

Third, although bladder temperature is equated with rectal temperature in the field of intensive care medicine, rectal temperature is mainly used in the context of forensic medicine to determine core body temperature. Results from bladder temperature measurements should be interpreted with caution.
